# The Cotton Centromere Contains a Ty3-*gypsy*-like LTR Retroelement

**DOI:** 10.1371/journal.pone.0035261

**Published:** 2012-04-19

**Authors:** Song Luo, Jennifer Mach, Bradley Abramson, Rolando Ramirez, Robert Schurr, Pierluigi Barone, Gregory Copenhaver, Otto Folkerts

**Affiliations:** 1 Chromatin, Inc., Chicago, Illinois, United States of America; 2 Department of Biology, The University of North Carolina at Chapel Hill, Chapel Hill, North Carolina, United States of America; 3 Carolina Center for Genome Sciences, The University of North Carolina at Chapel Hill, Chapel Hill, North Carolina, United States of America; 4 Chromatin, Inc., Champaign, Illinois, United States of America; Oklahoma Medical Research Foundation, United States of America

## Abstract

The centromere is a repeat-rich structure essential for chromosome segregation; with the long-term aim of understanding centromere structure and function, we set out to identify cotton centromere sequences. To isolate centromere-associated sequences from cotton, (*Gossypium hirsutum*) we surveyed tandem and dispersed repetitive DNA in the genus. Centromere-associated elements in other plants include tandem repeats and, in some cases, centromere-specific retroelements. Examination of cotton genomic survey sequences for tandem repeats yielded sequences that did not localize to the centromere. However, among the repetitive sequences we also identified a *gypsy*-like LTR retrotransposon (Centromere Retroelement *Gossypium*, CRG) that localizes to the centromere region of all chromosomes in domestic upland cotton, *Gossypium hirsutum*, the major commercially grown cotton. The location of the functional centromere was confirmed by immunostaining with antiserum to the centromere-specific histone CENH3, which co-localizes with CRG hybridization on metaphase mitotic chromosomes. *G. hirsutum* is an allotetraploid composed of A and D genomes and CRG is also present in the centromere regions of other AD cotton species. Furthermore, FISH and genomic dot blot hybridization revealed that CRG is found in D-genome diploid cotton species, but not in A-genome diploid species, indicating that this retroelement may have invaded the A-genome centromeres during allopolyploid formation and amplified during evolutionary history. CRG is also found in other diploid *Gossypium* species, including B and E2 genome species, but not in the C, E1, F, and G genome species tested. Isolation of this centromere-specific retrotransposon from *Gossypium* provides a probe for further understanding of centromere structure, and a tool for future engineering of centromere mini-chromosomes in this important crop species.

## Introduction

Centromeres in plants are typically gene poor, repeat rich regions that act as the site of kinetochore formation to link chromosomes to the microtubule spindle, thereby enabling chromosome segregation. At the cytological level, centromeres form a conspicuous constriction on metaphase chromosomes. The active centromere is marked by extensive epigenetic modification and deposition of a centromere-specific histone, CENH3 [1], [2]. Indeed, CENH3 deposition may be the epigenetic mark that specifies the functional centromere, as loss of CENH3 from dicentric chromosomes marks centromere inactivation, and deposition of CENH3 marks centromere reactivation or neo-centromere formation [3], [4]. Inactivation of centromeres and formation of centromeres in new locations indicate that the centromere DNA sequence is neither necessary nor sufficient for epigenetic centromere specification; however, reactivation of centromeres at their prior location indicates that the underlying sequence may provide a structurally suitable environment for deposition of epigenetic markers and centromere formation.

Although the epigenetic and cytoskeletal components of the centromere are conserved, the genomic sequences underlying the centromere vary substantially between species [5]. Many plants and animals have repetitive DNA in their centromeres, including multiple dispersed repetitive elements, and large blocks of direct, tandem repeats of a short sequence roughly the length of DNA that wraps around a nucleosome (reviewed in [6]). In plants, many centromere tandem repeats have been documented [6]; for example, the centromere tandem repeat is 156 nucleotides in maize [7], and 180 nucleotides in *Arabidopsis thaliana* [8]–[10]. Other economically important plant species with centromeric tandem repeats include sugarcane [11], sorghum [12], rice [13], wheat [14], [15], Brassica [16], *Beta* [17], Medicago [18], and soybean [19], [20].

In addition to tandem repeats, grasses (Poaceae) have centromere-specific Ty3-*gypsy* retroelements (REs) called CR elements [21]–[23], including CRM in maize [7] and CRR in rice [13]. In these species, CR elements tend to form clusters between large blocks of tandem repeats [24] and the tandem repeat regions of maize centromeres show very few insertions of other LTR REs [25]. Centromere-enriched REs have also been found in non-grass species, including soybean [20], beet [26], [17], Brassica [27] and tomato [28]. Analysis of maize centromere sequence suggests an important role for CRs in centromere evolution. Recently active CRM1 elements are located in the core of the active centromere, but other elements, such as CRM2, which have not been recently active, are located more peripherally, indicating that the centromere may have relocated [24]. Indeed, detailed examination of maize centromere sequence showed that CRM elements may act in removing tandem repeats [24].

Two lines of evidence indicate that centromere REs may be important for centromere function. First, CRM in maize and *cereba* in barley are bound by the centromere-specific histone CENH3 [29], [30], indicating that they are part of the functional centromere. Second, CRM and CRR elements produce transcripts that are thought to participate in small-RNA-mediated processes essential for centromere function [31], [32]. Centromere retroelements may also play an important role in the centromeres of species that lack tandem repeats; for example, the Drosophila centromere contains islands of complex DNA interspersed with long (approximately 100 kb), homogeneous stretches of simple satellite repeats (AATAT or AAGAG), more complex A+T rich repeats, and single, intact transposable elements [33], [34].

The presence of tandem repeats and the localization of centromere-specific REs have facilitated isolation of centromere sequences from many plants (reviewed in [5]). Nonetheless, our understanding of the role of these sequences in centromere function remains rudimentary at best. Also, the centromere sequence content of many important crop species, including those in the *Gossypium* genus, has not been described. To further our understanding of centromeres, we set out to examine the repetitive content of cotton centromeres. We identified a Ty3-*gypsy* LTR-retroelement, which we call CRG, that localizes to the centromere of all the chromosomes in domestic upland cotton, *Gossypium hirsutum*. *G. hirsutum* is an allotetraploid that combines A and D genomes [35]. The diploid *Gossypium* species have diverged into eight genome groups (designated by letters), including New World cottons (D genome type), Australian cottons (C, G, and K), and Asian-African cottons (A, B, E, and F). We show that CRG is present in diploid cotton species of the B, D, and E2 genome groups, but not in the A, C, E1, F, and G species tested, indicating that this element invaded the A genome centromeres from the D genome during allopolyploid formation.

## Results

### Identification of a cotton centromere retroelement

**Figure 1:**
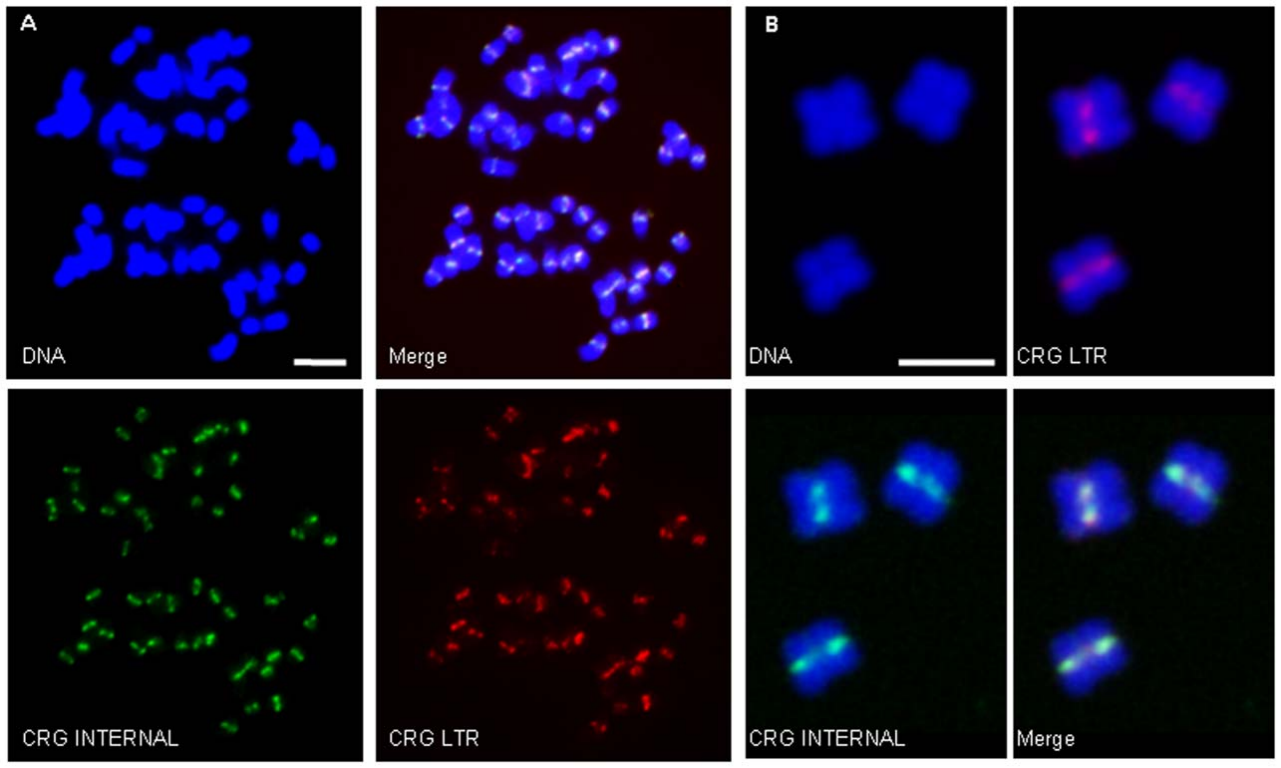
The CRG retroelement localizes to the centromere region in *G. hirsutum*. FISH using CRG1 sequences to probe mitotic chromosomes of cotton root tips. A. CRG1 hybridization (DAPI staining of DNA in blue, CRG1 internal sequences in green, CRG LTR in red). CRG1 is detected at a single locus on each of the chromosomes. Scale bar is 5 micrometers. B. CRG1 hybridization (DAPI staining of DNA in blue, CRG1 internal sequences in green, CRG LTR in red). CRG1 is detected at the primary constriction of cotton mitotic chromosomes. Scale bar is 2 µm.

To isolate sequences from the cotton centromere, we reasoned that if, like other plants, the cotton centromere comprises mainly repetitive DNA, then it should be over-represented in randomly generated cotton genomic survey sequence. To test this, we downloaded cotton genomic sequences from the public databases and assembled them into contigs using low stringency parameters (see Methods). The contigs containing the most overlapping sequences (the “deepest reads”) were selected for further analysis. The sequences with the deepest sequence coverage were grouped into classes by sequence similarity and a representative sample from each class was used as a FISH probe to determine possible localization to the cytological centromere. This approach identified one contig containing an LTR retrotransposon (see below) that specifically hybridizes to the primary constriction of all 52 chromosomes in *Gossypium hirsutum* TM-1 (Figure 1). Following the nomenclature of other centromere-associated elements, we have designated this element Centromere Retroelement *Gossypium*, (CRG).

### CRG localizes in or near the functional centromere, as marked by CENH3

**Figure 2:**
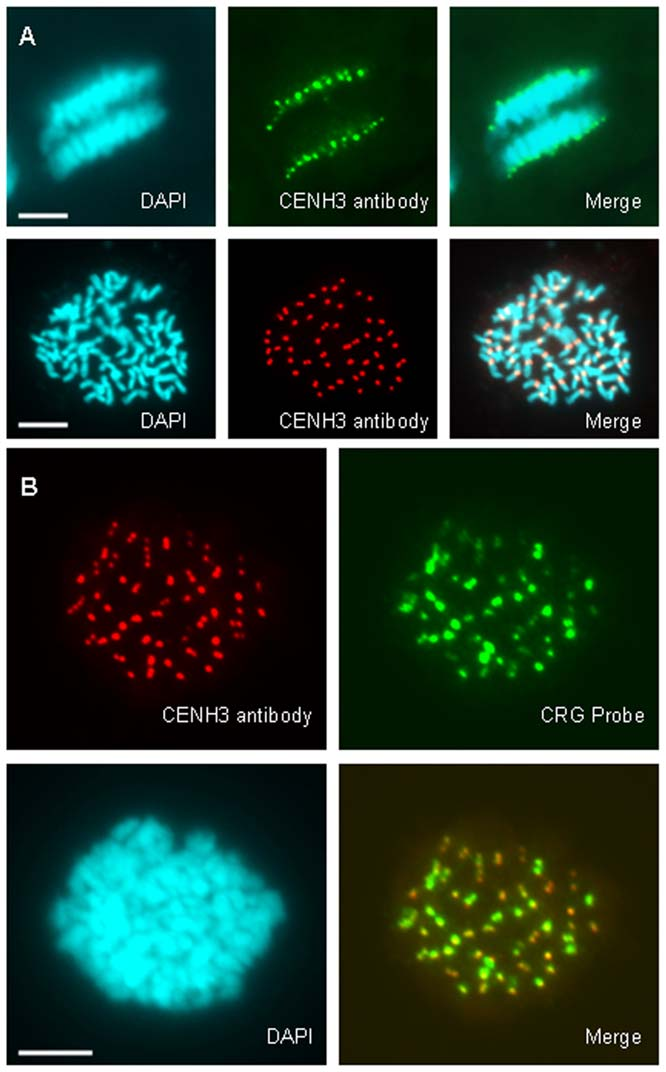
The CRG retroelement co-localizes with CENH3 immunostaining. A. Immunostaining using anti-CENH3 antiserum on cotton mitotic chromosomes. Top, DAPI-stained chromosomes (blue) at anaphase are stained with anti-CENH3 (green), which localizes to the centromere region. Bottom, DAPI-stained chromosomes (blue) at metaphase are stained with anti-CENH3 (red), which localizes to the centromere region. B. Co-localization of anti-CENH3 immunostaining (red) and FISH signal with CRG1 probe (green) on cotton mitotic metaphase chromosomes (blue). Scale bar is 5 µm in A and B.

To determine whether CRG localization to the primary constriction represents localization to the region of the functional centromere, we confirmed the location of the centromere by immunostaining with antibodies to the centromere-specific histone CENH3. We first identified cotton *CENH3* by searching for sequences similar to histone H3 in the cotton EST database, using the TBLASTN algorithm. CENH3 proteins generally have a divergent N-terminal tail, and higher sequence similarity to the non-centromeric histone H3 in the C-terminal histone core [36]. Therefore, we selected sequences with 40 to 70% amino acid sequence identity as good candidates for further characterization. By aligning the predicted amino acid sequences with CENH3 from different species, we found two good EST candidates for cotton *CENH3* (DT566672 and DR460547). These two sequences share greater than 98% identity at both the DNA and predicted amino acid sequence level, but DT566672 is a full-length cDNA, and DR460547 is only a partial sequence. We used the first 18 amino acids at the N-terminus, which are predicted to be distinct from H3, to produce a polyclonal anti-peptide antiserum. We then used this antiserum to immunostain spreads of cotton mitotic chromosomes, and detected staining with a fluorescent secondary antibody. Pre-immune controls showed no staining, but immunofluorescence results using the anti-peptide antiserum clearly showed centromere staining for every chromosome (Figure 2A), consistent with the hypothesis that this antibody specifically detects the centromere marker CENH3.

To assess whether CRG localizes to the functional centromere, we next examined whether the immunofluorescence signal from CENH3 colocalized with the FISH signal for CRG. In the combined FISH and immunofluorescence staining, we found that CRG and CENH3 stained the same location, the primary constriction, on each cotton chromosome (Figure 2B). Therefore, this result shows that CRG sequences colocalize with the functional centromere of cotton. Because metaphase chromosomes are highly condensed, this staining cannot provide precise co-localization information and further high-resolution studies such as fiber-FISH or chromatin immunoprecipitation (ChIP) will be required to determine whether CRG is present in the functional centromere.

### CRG, a Ty3-gypsy element

Our analysis identified two CRG elements, a shorter element of 4,800 nucleotides, including 2,836 bp of internal sequence between the 982-nucleotide LTRs (CRG1; JQ009328), and a long CRG (CRG2; JQ009329) of 10,911 nucleotides with an internal sequence also flanked by LTRs (Figure S1). Comparison to previously isolated sequences by BLAST indicated that these elements are most closely related to the *gypsy*-like class of plant retroelements. Examination of the CRG1 and 2 sequences with Genescan (http://genes.mit.edu/GENSCAN.html) predicted peptides of 589 and 1138 amino acids from CRG1 and 2, respectively (Figure S1). CRG2 showed typical retroelement domains consistent with a *gypsy*-like retroelement (LTR-*gag*-protease-RT-RNaseH-IN-LTR) [22]. The two LTRs of CRG2 are only 92% identical, indicating that this element was a more ancient insertion than the short element, which has LTRs that are 100% identical [37]. The short element does not contain a complete polyprotein-encoding region, indicating that it may transpose non-autonomously. Sequence searches also identified cotton expressed sequence tags (ESTs) with high sequence similarity to the CRG LTRs. Thus, some CRG elements may be active and some may be inactive, or transpose non-autonomously.

### The CRG element is present in AD, B, D, and E2 genome species, but not in A, C, E1, F, and G genome species

**Figure 3:**
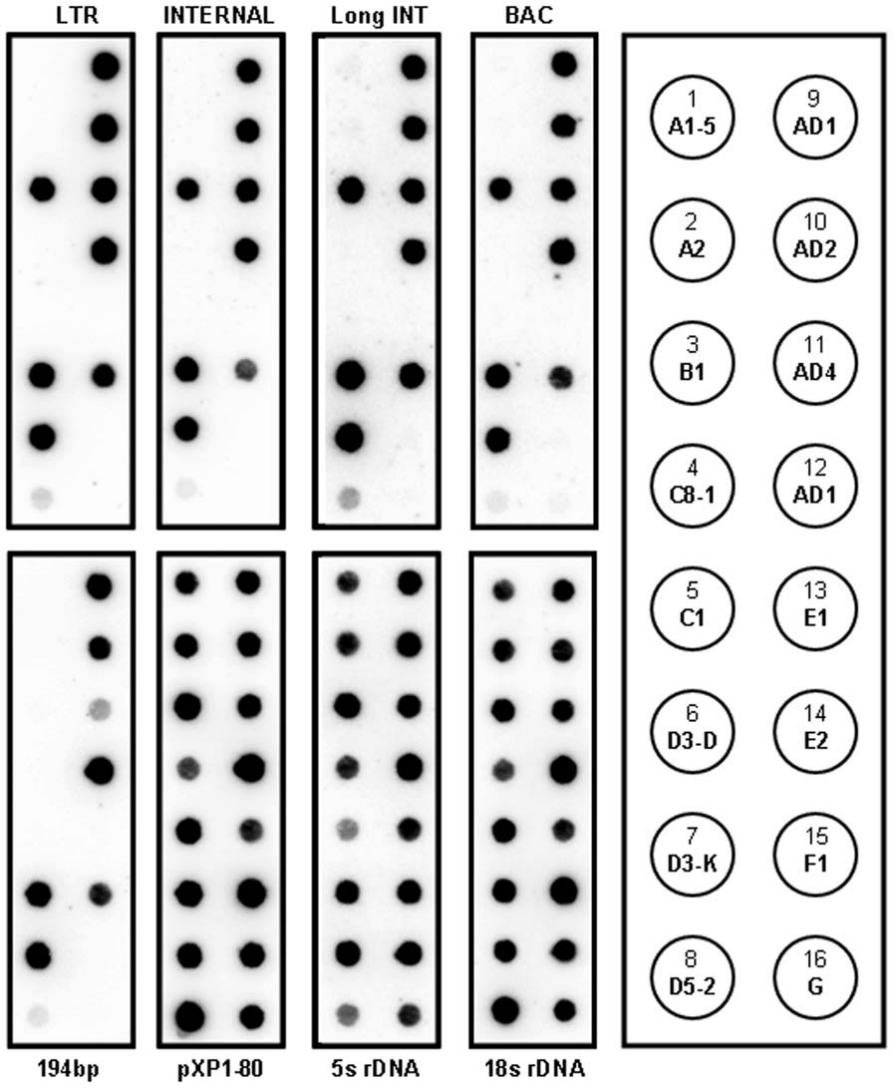
CRG elements are present in other *Gossypium* species. DNA dot blots of genomic DNA from diploid and tetraploid *Gossypium* species representing the indicated genomic groups (key, right panel with dots numbered sequentially and genome type indicated, see below) were hybridized with the indicated probes. Probes: LTR, CRG1 LTR; INTERNAL, non-LTR internal sequence for short CRG1 element; longINT, non-LTR sequence for long CRG2 element; BAC, centromere-localized BAC Gh53H10; 194, 194-nt tandem repeat; pXP1-80; 5S rDNA; 18S rDNA. Species: 1, *G. herbaceum* (A1); 2, *G. arboreum* (A2); 3, *G. anomalum* (B1); 4 *G. pulchellum* (C8); 5, *G. nandewarense* (C1); 6, *G. davidsonii* (D3-D); 7, *G. klotzschianum* (D3-K); 8, *G. raimondii* (D5-2); 9, *G. hirsutum* TX 61 (AD1); 10, *G. barbadense* (AD2); 11, *G. mustelinum* (AD4); 12, *G. hirsutum* cultivar TM-1 (AD1); 13, *G. stocksii* (E1); 14, *G. somalense* (E2); 15, *G. longicalyx* (F1); 16, *G. nelsonii* (G).

*G. hirsutum* is a tetraploid derived from a recent allopolyploidization event, which brought together a New World D genome and an African-Asian A genome approximately 1 million years ago [35]. To test whether the CRG element may have been present in the centromere regions of the progenitor diploid *Gossypium* A and D genomes, we used dot blot hybridization and FISH with CRG1 sequences to verify CRG presence and localization in existing A and D diploid species. We found that the CRG element is present in the three D genome species we examined (*G. davidsonii* D3-3, *G. klotzschianum* D3-K, *G. raimondii* D5-2; (Figure 3). Sequences highly similar to the CRG element were also found by BLAST searches in the *G. raimondii* D genome genomic survey sequences, confirming the dot blot results. In these species, the CRG element also localizes to the centromere region (Figure S2). However, no hybridization was detected, either by dot blot, or by FISH, in the A genome species tested (*G. herbaceum* A1-5, *G. arboreum*, A2, Figure 3 and Figure S2).

To determine how widespread the occurrence of the CRG element is in other *Gossypium* genomes, we examined other diploid cotton species by the same methods (Figure 3 and Figure S2). Intriguingly, although the CRG element was not present in the A genome species tested, it was found in the centromere regions of two other African-Asian species, the B genome *G. anomalum* and the E2 genome *G. somalense*, but not in the E1 genome *G. stocksii*. The F genome African-Asian species (*G. longicalyx*) was also negative, as were three Australian species tested (*G. nandewarense*, C1, *G. pulchellum* C8-1, and *G. nelsonii*, G). These results are summarized in Table 1. Thus, the CRG element is present in both New World and Old World lineages of diploid cottons, but absent in Australian and some Old World lineages.

**Table 1:** Repeat content of *Gossypium* species.

Species		CRG short internal	CRG long internal	CRG LTR	CRG BAC 53H10	194 bp repeat	pXP 1–80	5S rDNA	18S rDNA
*G. herbaceum*	A1-5	−	−	−	−	−	+	+	+
*G. arboreum*	A2	−	−	−	−	−	+	+	+
*G. anomalum*	B1	+	+	+	+/−	−	++	+	+
*G. pulchellum*	C8-1	−	−	−	−	−	+	+	+
*G. nandewarense*	C1	−	−	−	−	−	+	+/−	+
*G. davidsonii*	D3-3	+	+	+	+	+	+	+	+
*G. klotzschianum*	D3-K	+	+	+	+	+	+	+	+
*G. raimondii*	D5-2	+/−	+/−	+/−	+/−	+/−	++	+/−	++
*G. hirsutum*	AD1	+	+	+	+	+	+	+	+
*G. barbadense*	AD2	+	+	+	+/−	+	+	+	+
*G. mustelinum*	AD4	+	+	+	+	+/−	+	+	+
*G. hirsutum TM-1*	AD1	+	+	+	+	+	++	+	++
*G. stocksii*	E1	−	−	−	−	−	+	+	+
*G. somalense*	E2	+	+	+/−	+/−	+	++	+	++
*G. longicalyx*	F1	−	−	−	−	−	+	+	+
*G. nelsonii*	G	−	−	−	−	−	+	+	+

Summary of hybridization signals identified by dot blot analysis (Figure 3). Hybridization signals ranged from strong (++), moderate (+), and weak (+/−) to absent (−).

### Sequences flanking the CRG elements from the AD genome identify the centromeres of the non-CRG-containing *Gossypium* species

**Figure 4:**
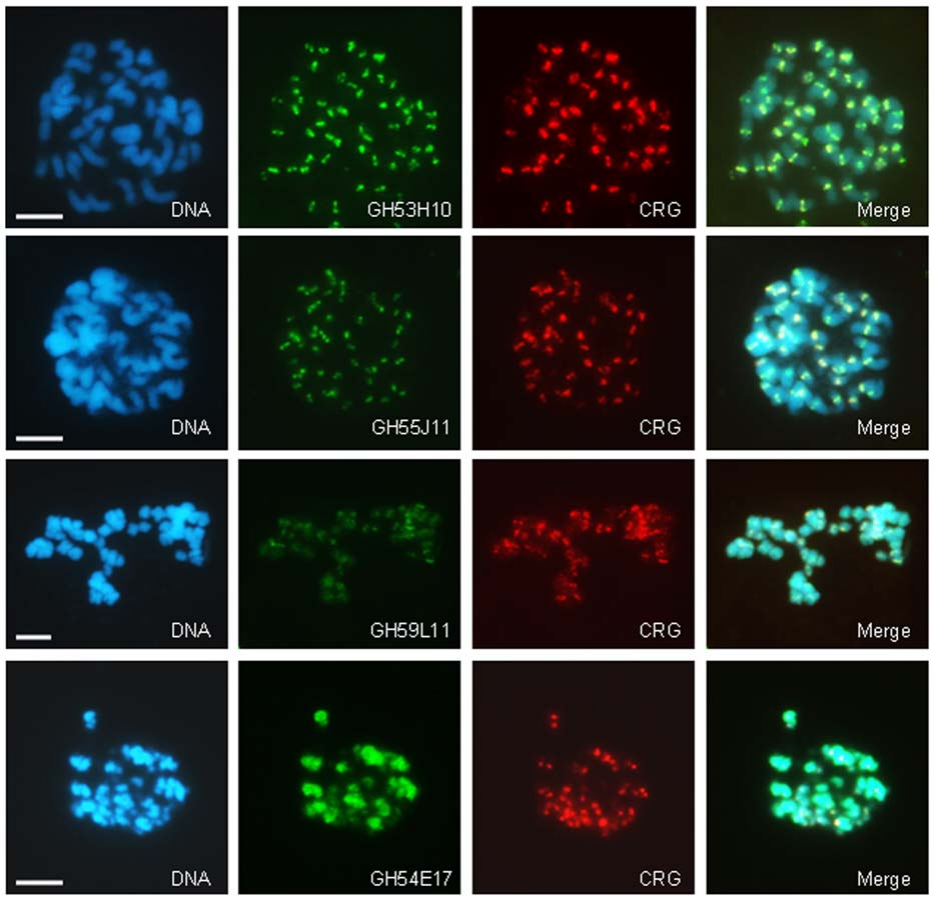
Sequences adjacent to CRG in *Gossypium hirsutum* are centromere-specific, pericentromeric, or heterochromatic. DAPI-stained mitotic metaphase cotton chromosomes (blue) from *G. hirsutum*, were hybridized with different CRG-containing BAC sequences (green), as indicated, and with the CRG1 element (red). GH53H10 and GH55J11 have strong and specific centromere localization, but other BACs detect more dispersed or pericentromeric sequences. Scale bar is 5 µm.

Examination of sequences adjacent to CRG will help us understand the genomic context of CRGs across the cotton genome and within the centromere. To isolate these sequences, we used the CRG element as a probe to identify bacterial artificial chromosome (BAC) clones from a library derived from the AD genome species *G. hirsutum*. We then used FISH to examine the genomic localization of these BACs in the AD genome. Some BACs showed strong and specific centromere hybridization and others showed more diffuse centromere hybridization or heterochromatin localization (Figure 4). Thus, this shows that some sequences adjacent to CRGs in the genome are specific to the centromere, but some adjoining sequences are peri-centromeric or heterochromatic. This latter class likely contains dispersed repetitive elements that are present both in the centromere and the heterochromatin.

**Figure 5:**
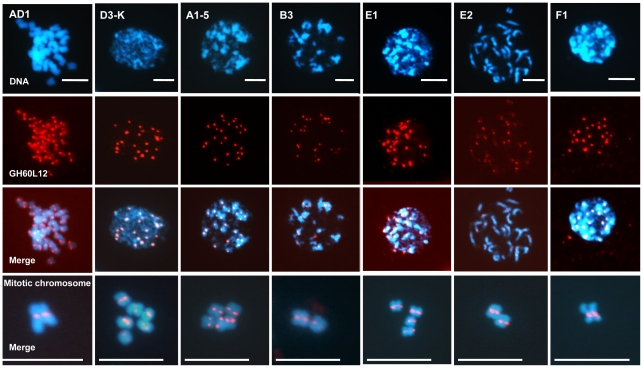
Sequences flanking the CRG elements from the AD genome hybridize to the centromeres of the non-CRG-containing *Gossypium* genomes. DAPI-stained cotton interphase or mitotic nuclei (blue) from *Gossypium* species, as indicated, were hybridized with a CRG-containing BAC, GH60L12 (red), which shows localization to the centromere region in the tested *Gossypium* species, including those species that do not contain the CRG element in their genomes (A, E1, and F1). Interphase spreads (top panels) show strong foci of the correct number, consistent with centromere localization. Also, metaphase chromosomes (bottom panels) show localization to the primary constriction.

We further used the centromere-specific BACs to examine the centromeres of the diploid cotton species that do not contain CRG. We hypothesized that if the CRG element colonized the A genome from the D genome, then sequences flanking the CRG elements in the AD genome may identify the centromeres of the genomes that do not contain the CRG element. We hybridized one of the centromere-specific BACs to chromosomes from cotton species representing the different genome types (Figure 5 and Figure S3). This BAC specifically detected the *Gossypium* genome centromeres in all genome types tested, including those that did not hybridize to the CRG element, indicating the presence of non-CRG conserved sequences in cotton centromeres. For example, in the A-genome species, BAC GH60L12 hybridizes to centromere regions, even though this species does not contain the CRG element. Thus, centromere-region hybridization in A-genome species is likely mediated by non-CRG sequences that are present, and possibly conserved, in the cotton centromere.

### Tandem Repeats

**Figure 6:**
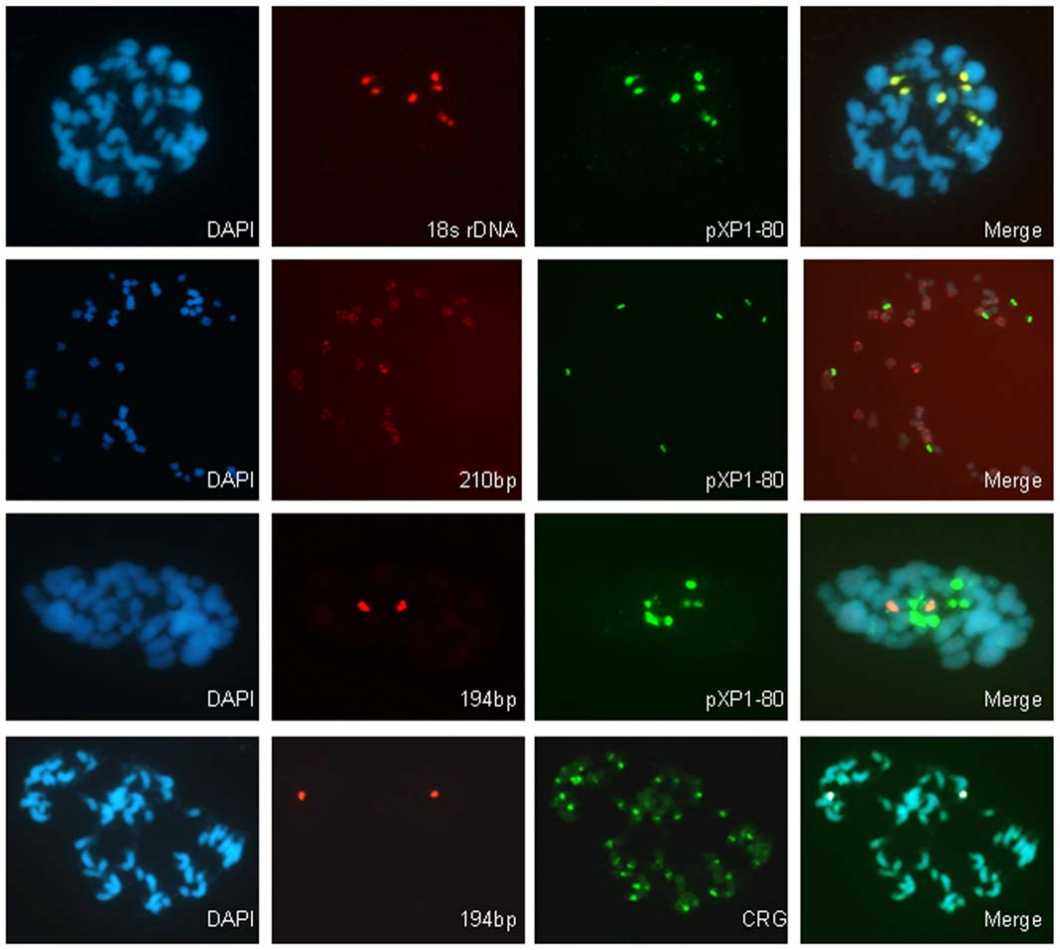
Tandem repeats localize to non-centromeric regions in *G. hirsutum*. DAPI-stained mitotic metaphase cotton chromosomes (left panels, blue) were hybridized with FISH probes for two different tandem repeats (center panels, red and green). The merged images show whether the repeats localize to the centromere, or colocalize with other genomic markers.

Because tandem repeats are associated with the centromere in many species, we also used tandem repeats as FISH probes to query the cotton genome (Figure 6). The tandem repeats were selected from those previously described in the literature, or by bioinformatic analysis of cotton genomic sequences to find simple tandem repeats (see Methods: 210-bp tandem repeat (JQ009325), 100-bp tandem repeat (JQ009326) and 194-bp tandem repeat (JQ009327)). None of the repeats examined displayed obvious localization to the cytological centromere. For example, a 194-bp repeat identified from genomic sequences localizes near the centromere of a single chromosome, but not on any other chromosome. The pXP1-80 (AF060649.1) repetitive element shares many of the characteristics of a centromere repeat, in that it is a tandem repeat present in all tested cotton species and is similar in size to other known centromere repeats [38]. However, we found that it does not localize to the centromere but instead co-localizes with an 18S rDNA probe (Figure 6).

## Discussion

Here we have identified a retroelement, CRG, which localizes to the centromere region of *Gossypium* species. CRG is present in the centromere region of in all chromosomes in the AD genome tetraploid domestic cotton *G. hirsutum*, and in D-genome diploid species. However, CRG is not present in the A genome species tested, indicating that the A genome centromeres were invaded by CRG elements in the tetraploid. This centromere localization following active transposition indicates that CRG may transpose specifically into centromere sequences. LTR retroelements move by replicative transposition, wherein the parent element produces copies that integrate in other genomic locations [39]. These genomic locations may be random, or may show some specificity; for example, the maize centromere retroelement CRM seems to specifically target the functional centromere [24]. In addition to replicative transposition, gene conversion may also affect the distribution of CRG elements, as gene conversion between homologous chromosomes has recently been documented in the maize centromere core [40].

The presence of CRG elements in the A centromere regions of AD tetraploids indicates that CRG elements can invade new centromeres. Also, the presence of CRG sequences among cotton ESTs and the high sequence similarity between the two LTR sequences of the same CRG indicate that CRG elements may actively transpose. To limit genomic damage, plant hosts have evolved RNA interference-meditated mechanisms to tame their endogenous retroelements [41]; these mechanisms may have been interrupted during polyploid formation, allowing a burst of CRG transposon activity. Although many examples of transposon activation following hybridization or polyploid formation have been seen (for example, [42], [43]), recent work indicates that cotton retroelements, including the gypsy-like GORGE3, did not show a burst of activity after allopolyploid formation [44]. Indeed, activation of transposition in polyploid formation, as seen for CRG, may be the exception rather than the rule [45].

While invading the A chromosomes, the CRG elements may also have proliferated on the D chromosomes in the AD species, as indicated by the relatively low signal intensity for CRG in the D genome species, *G. raimondii*, compared to the AD genome species. In a polyploid, movement of repetitive sequences between the two genomes is not unprecedented; for example, a FISH analysis of diploid and tetraploid cotton species showed that many (17 out of 20 tested) dispersed repetitive sequences from the A genome have spread to the D genome [46], [47]. However, in these studies, spread in the D to A direction was rare. In addition to replicative transposition, gene conversion between homoeologous chromosomes presents another possible mechanism for CRG spread and elimination.

The identification of a retroelement that localizes to the centromere regions of *G. hirsutum* centromeres frames two intriguing questions for future work. First, does the cotton centromere contain a tandem repeat? Using this marker, and published sequences, we find no evidence of a centromeric tandem repeat in cotton. However, rice centromere sequences show that the tandem repeat array at a functional centromere can be as small as 65 kb [48]. Additional studies, including complete sequencing of cotton centromeres, will be required to unambiguously determine whether the cotton centromere contains a tandem repeat. Although it is not possible to exclude the presence of a tandem repeat at this time, it may be that in cotton, the functional centromere is defined by the presence of a retroelement without interspersed tandem repeats.

Our results also prompt a second question: do CRG elements contribute to centromere function in cotton? A key consideration is whether CRG is part of the functional centromere, as determined by high-resolution methods such as fiber-FISH and ChIP. CRG is not present in the centromeres of some diploid cotton genomes; therefore, CRG is not essential for centromere function in all cotton species. However, CRG is embedded in sequences that are present in all cotton centromeres (Figure 5). If CRG marks the functional cotton centromere, then an additional question is whether CRG-containing sequences can provide a suitable site for CENH3 deposition and centromere formation. Although epigenetic mechanisms, specifically CENH3 deposition, specify the centromere, work in plants and animals indicates a role for underlying DNA sequences in producing a suitable structure or environment for CENH3 deposition. Comprehensive functional testing via formation of engineered mini-chromosomes will be useful in addressing this issue. Identification of CRG provides a valuable entry into centromere structure and function, and has helped identify centromere sequences that may be useful for construction of centromere-based mini-chromosome vectors for gene stacking applications in cotton biotechnology.

## Methods

### Cotton varieties

Cotton cultivars were acquired from the National Plant Germplasm System (http://www.ars-grin.gov/npgs/index.html) under the following accession numbers: *G. herbaceum* (PI 175456), *G. arboreum* (PI 183160), *G. anomalum* (PI 530743), *G. pulchellum* (PI 464858), *G. nandewarense* (PI 530752), *G. davidsonii* (PI 530809), *G. klotzschianum* (PI 530882), *G. raimondii* (PI 530899), *G. hirsutum* TX 61 (PI 154094), *G. barbadense* (PI 407497), *G. mustelinum* (AD4 9), *G. hirsutum* cultivar TM-1 (PI 607172), *G. stocksii* (PI 530976), *G. somalense* (PI 530890); 15, *G. longicalyx* (PI 530986); 16, *G. nelsonii* (PI 530763). Cotton plants were grown in a greenhouse (16-h d, 26–28°C) in 1.6-gallon pots containing 1∶1∶1 soil∶ peat∶ perlite.

### Bioinformatics Methods

To find sequence contigs with deep reads, 49,906 genomic survey sequences from *Gossypium* were downloaded from Genbank GSS database. Using Phrap program version 0.990329 [49], a first assembly was performed with no vector cleaning but with high stringency parameters to align the GSS sequences [49], in order to form reliable contigs on the assembly draft. All singletons were ignored and only contigs that were obtained from the first assembly draft were used in the second assembly. The parameters used this time were less stringent. Using the program Tandem Repeat Finder (http://tandem.bu.edu/trf/trf.html) [50], these contigs were examined for tandem repeats. Tandem repeats were identified using criteria as described [51]. To identify contigs with deep reads, we used the .ACE format file produced by Phrap on the second assembly and found 14 sequences containing more than 75 reads per contig. The visual analytical tool Hawkeye [52] was used to provide a sorting method to cluster contigs with the highest numbers of reads from the second assembly; these contigs were used for further analysis. The CRG1 and 2 peptides were found using Genescan (http://genes.mit.edu/GENSCAN.html).

### Fluorescence *In Situ* Hybridization

FISH on cotton root tips essentially followed the published protocol [53]. Briefly, excised root tips were arrested for 3 hours at 150 pounds/square inch nitrous oxide and fixed in 90% acetic acid. Root meristems were excised, transferred to citrate buffer (10 mM sodium citrate/citric acid, pH 4.8), and digested with 5% cellulase (Calbiochem) and 1% macerozyme (Calbiochem) for 1.5–2 hours. Digested root meristems were washed with TE (10 mM Tris, 1 mM EDTA, pH 8.0) containing 100 ug/ml RNaseA (Invitrogen, Carlsbad, CA), then cold 100% ethanol. The tissue was disrupted and resuspended in 100% acetic acid (use 15 µl per root tip for each slide), then pipetted onto a poly-L-lysine coated slide (Polysciences, Inc.), dried in a semi-humid chamber, and crosslinked (optimized setting, Spectrolinker XL-1000, Spectronics Corp.).

Probes were labeled with ChromaTide Alexa Fluor 488 or 568 dyes (Invitrogen) by nick translation and column purified (Qiagen), following the manufacturer's instructions. Slides were washed in 2× SSC, and immediately hybridized in 52.5% formamide, 2.3× SSC, 10.5% dextran sulphate, and 0.12 mg/ml salmon sperm DNA, with approximately 20 ng labeled probe and denatured on a PCR machine block at 85°C (90 seconds), then 70°C (30 sec.), 60°C (30 sec.), 50°C (30 sec.), 37°C (30 sec.), then incubated at 37°C in a humid chamber overnight. Slides were washed in 2× SSC at 55°C for 20 minutes, then mounted in Vectashield with DAPI (Vector Laboratories, Burlingame CA). Slides were imaged using a Zeiss LSM 710 Confocal Microscope or Nikon Eclipse E800 fluorescence microscope.

### Immunofluorescence

The anti-CENH3 antiserum was produced in guinea pigs using synthetic peptide from the cotton CENH3 N-terminal sequence N-MSRTKHTAAKKPRRKPSA (Covance, Inc.). Immunostaining was performed as described [54] with minor modifications. Root tips were harvested from actively growing seedlings and fixed with 4% paraformaldehyde in PHEMES (0.06 M PIPES, 0.025 M HEPES, 0.01 M EGTA, 2 mM MgCl_2_, 0.3 mM sorbitol, pH 6.8) for 20 min. After being washed with 1× PBS (0.01 M NaH_2_PO_4_, 0.14 M NaCl, pH 7.0), the root tips were squashed onto a slide without any prior cellulase or pectinase treatment. Approximately 100 µl guinea pig anti- GhCENH3 antibody diluted 1∶200 in TNB buffer [0.1 M Tris- HCl, 0.15 M NaCl, pH 7.5, with 0.5% blocking reagent (Sigma)] was then added, and after incubation in a humid chamber at 37°C for 3 h, the slides were washed 3× in 1× PBS before the addition of 100 µl FITC or TRITC - conjugated anti-guinea pig secondary antibody (Sigma; 1∶200 in TNB buffer). Incubation and washes were as for the primary antibody. The slides were counter-stained with 4′,6-diamidino-2- phenylindole (DAPI) prior to microscopy.

### DNA Dot blots

DNA was prepared from cotton leaves using hexadecyltrimethylammonium bromide [55] and the concentration was quantified using Picogreen (Invitrogen) according to the manufacturer's instructions. For the dot blot, 0.5 µg of DNA for each species was spotted onto a nylon membrane (Amersham Hybond XL, GE Healthcare) and crosslinked (Spectrolinker XL-1000, Spectronics Corp). Probes were prepared by PCR (primers are listed in Table S1) and labeled with the Amersham Rediprime II system (GE Healthcare). Blots were hybridized in 5× SSC, 0.5% SDS, 25 mM sodium phosphate, 5× dextran sulphate, and 0.2 mg/ml salmon sperm DNA, and washed in 0.5× SSC, 0.5% SDS at 65°C for 3 hours. The signal was imaged using a phosphorimager (Amersham Storm 860, GE Healthcare). For re-probing, blots were stripped in 0.1M NaOH, 10 mM EDTA, 0.1% SDS for 30 minutes at room temperature, then washed in 0.1× SSC, 0.1%SDS, 0.2M Tris pH 7.5 for 30 minutes. Efficacy of stripping was confirmed by phosphorimaging.

## Supporting Information

Figure S1 **Diagram of CRG structure.** CRG retroelement structure is shown to scale (1 kb = 0.5 inch), with Long Terminal Repeats (LTRs) shown as blue arrows and core sequence shown as a line. The predicted coding regions of the two CRGs are shown as red lines, with conserved domains (G, GAG; P, Protease; R, Reverse Transcriptase; H, RNAseH; I, Integrase) presented as bars. The two CRG1 LTRs are 100% identical. The green line indicates the region conserved between the two CRG elements. The asterisk indicates 480 nucleotides of sequence that is missing from the CRG2 sequence.
(TIF)

Figure S2 **In other** ***Gossypium*** **species, the CRG element also shows foci, consistent with localization to the centromere region.** DAPI-stained cotton nuclei (blue) from different *Gossypium* species, as indicated, were hybridized with FISH probes for the CRG1 element (green) and the 18S ribosomal DNA (red).
(TIF)

Figure S3 **In other** ***Gossypium*** **species, the CRG element also localizes to the centromere region.** DAPI-stained mitotic metaphase cotton chromosomes (blue) from *Gossypium* species, as indicated, were hybridized with a CRG-containing BAC, GH60L12 (red), which shows strong centromere localization in the tested *Gossypium* species, including those that do not contain the CRG element.
(TIF)

Table S1 **Primers used in this study.** PCR primers used to amplify specific repeats or sequences in this study, and their sequences are listed.
(DOCX)
