# Supplementary figures and images for: The Cotton Centromere Contains a Ty3-gypsy-like LTR Retroelement
Source: PLoS One. 2012 Apr 19;7(4):e35261. doi: 10.1371/journal.pone.0035261 (PMC3334964; doi:10.1371/journal.pone.0035261)

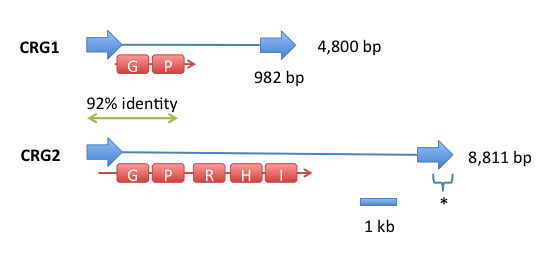

Supplement: Figure S1 — Diagram of CRG structure. CRG retroelement structure is shown to scale (1 kb = 0.5 inch), with Long Terminal Repeats (LTRs) shown as blue arrows and core sequence shown as a line. The predicted coding regions of the two CRGs are shown as red lines, with conserved domains (G, GAG; P, Protease; R, Reverse Transcriptase; H, RNAseH; I, Integrase) presented as bars. The two CRG1 LTRs are 100% identical. The green line indicates the region conserved between the two CRG elements. The asterisk indicates 480 nucleotides of sequence that is missing from the CRG2 sequence. (TIF) [file pone.0035261.s001.tif]

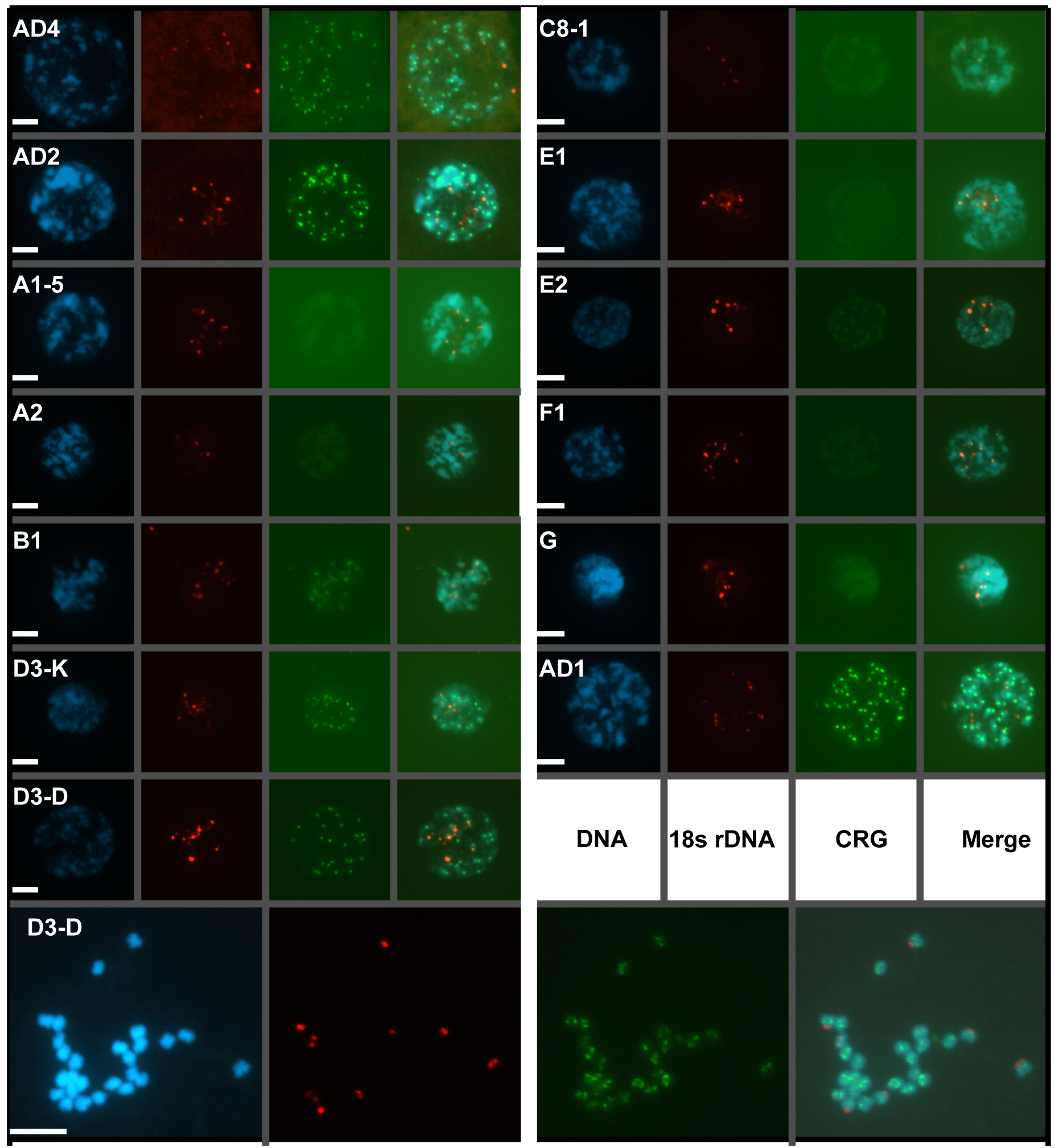

Supplement: Figure S2 — In other Gossypium species, the CRG element also shows foci, consistent with localization to the centromere region. DAPI-stained cotton nuclei (blue) from different Gossypium species, as indicated, were hybridized with FISH probes for the CRG1 element (green) and the 18S ribosomal DNA (red). (TIF) [file pone.0035261.s002.tif]

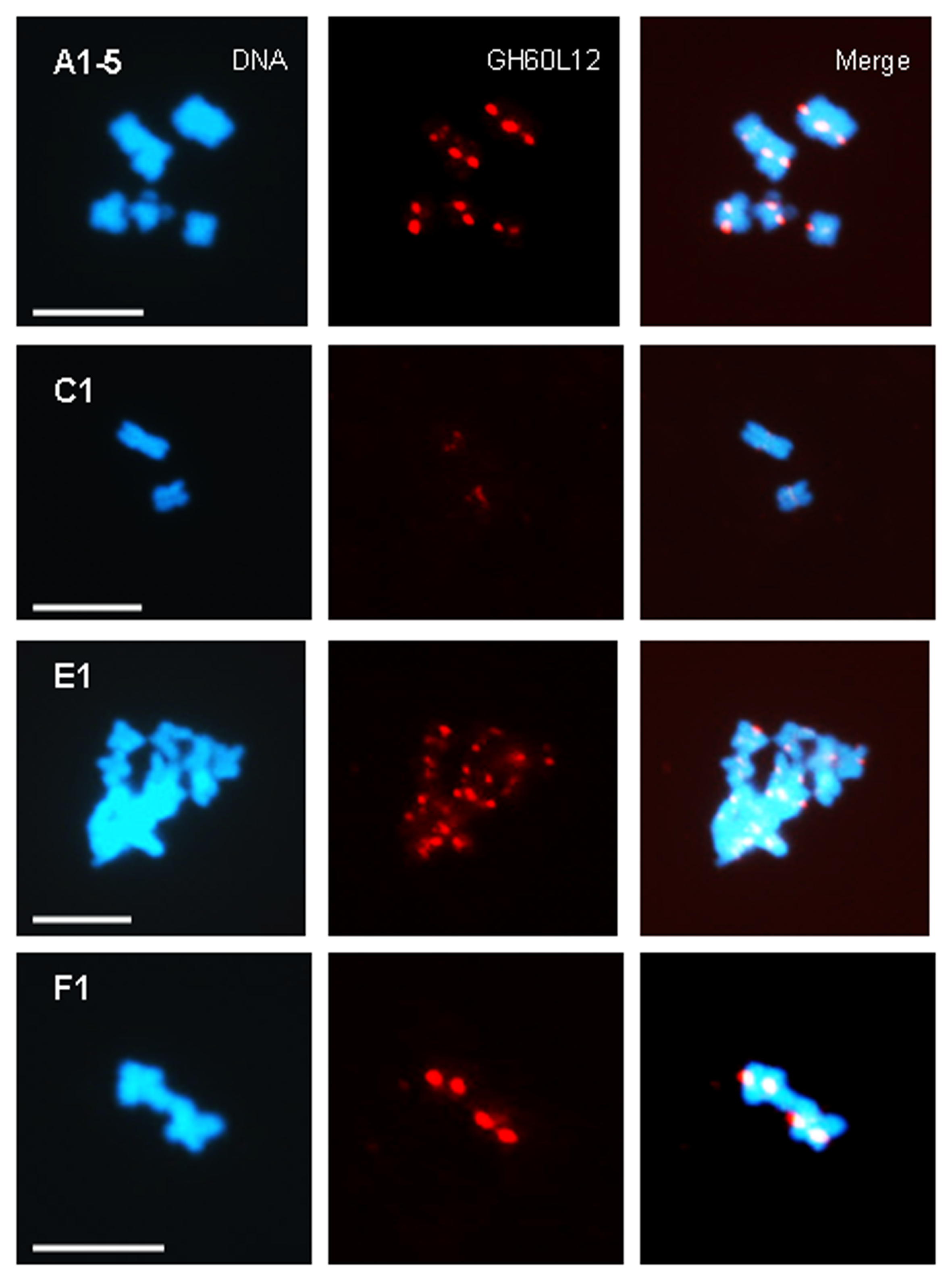

Supplement: Figure S3 — In other Gossypium species, the CRG element also localizes to the centromere region. DAPI-stained mitotic metaphase cotton chromosomes (blue) from Gossypium species, as indicated, were hybridized with a CRG-containing BAC, GH60L12 (red), which shows strong centromere localization in the tested Gossypium species, including those that do not contain the CRG element. (TIF) [file pone.0035261.s003.tif]
