# Supplementary material for: The Cotton Centromere Contains a Ty3-gypsy-like LTR Retroelement
Source: PLoS One. 2012 Apr 19;7(4):e35261. doi: 10.1371/journal.pone.0035261 (PMC3334964; doi:10.1371/journal.pone.0035261)
Supplement: Table S1 — Primers used in this study. PCR primers used to amplify specific repeats or sequences in this study, and their sequences are listed. (DOCX) [file pone.0035261.s004.docx]

Table S1: Primers used in this study

| Target sequence | Primer sequence 5’-3’ |
| --- | --- |
| 194bp satellite forward | GAGTCTTGGGGCATTGAAAC |
| 194bp satellite reverse | GAGCTAGTTGGGCTCGCTAT |
| 210bp satellite forward | AGTGGAAATGCCAGACTTCG |
| 210bp satellite reverse | GCTGATTTGGCTTTCATGTG |
| 100bp satellite forward | CCCCGACATTGCAGTTAAAA |
| 100bp satellite reverse | TGGGGAGTAAGATTCGCTGT |
| cotton pXP1-80 forward | CGAGGCTCCCGCACATCCAA |
| cotton pXP1-80 reverse | GGCACCATCGGCACCTAGG |
| cotton 18s rDNA forward | AAACGGCTACCACATCCAAG |
| cotton 18s rDNA reverse | GTACAAAGGGCAGGGACGTA |
| CRG forward | CAAATTACCATTTTTCAATGATGTTGG |
| CRG reverse | AAACTGAGGGCAGATGATGGTTTA |
| CRG LTR forward | CACGGGAAACTCCTACGAAA |
| CRG LTR reverse | CGAGTCACAAAAGCCCAAAT |
| CRG core forward | CCCCCTTCCTCAAAAAGATT |
| CRG core reverse | ATCAGATTTTGGGCGTTTTGG |
|  |  |
